# Migration drivers and migration choice: interrogating responses to migration and development interventions in West Africa

**DOI:** 10.1186/s40878-022-00283-3

**Published:** 2022-03-07

**Authors:** Richard Black, Alice Bellagamba, Ester Botta, Ebrima Ceesay, Dramane Cissokho, Michelle Engeler, Audrey Lenoël, Christina Oelgemöller, Bruno Riccio, Papa Sakho, Abdoulaye Wotem Somparé, Elia Vitturini, Guido Nicolas Zingari

**Affiliations:** 1 College of Social Sciences, University of Birmingham, Edgbaston, B15 2TT UK; 2 Università Degli Studi Di Milano-Bicocca, Milan, Italy; 3 Université Kofi Annan, Conakry, Guinea; 4 Université Cheikh Anta Diop, Dakar, Senegal; 5 Universität Basel, Basel, Switzerland; 6 CNRS, Institut Convergences Migrations, Paris, France; 7 Loughborough University, Loughborough, UK; 8 Università Di Bologna, Bologna, Italy; 9 Università di Torino, Turin, Italy

**Keywords:** Migration, Development, Choice, Uncertainty, West Africa

## Abstract

The notion of migration as being at least partly about ‘choice’ is deeply rooted in both academic thought and public policy. Recent contributions have considered migration choice as step-wise in nature, involving a separation between ‘aspiration’ and ‘ability’ to migrate, whilst stressing a range of non-economic factors that influence migration choices. But such nuances have not prevented the emergence of a significant area of public policy that seeks to influence choices to migrate from Africa through ‘irregular’ channels, or at all, through a range of development interventions. This paper explores evidence from West Africa on how young people formulate the boundaries of such choice. Drawing on approaches in anthropology and elsewhere that stress the value of a ‘future-orientated’ lens, we show how present uncertainty is a central framing that fundamentally limits the value of thinking about migration as a choice. This has important implications for policy on ‘migration and development’.

## Introduction

Debate about the causes, or ‘drivers’ of migration is longstanding, and critiques of false dichotomies—‘voluntary’ vs. ‘forced’, for example, or ‘economic’ vs. ‘political’—are numerous. Migration scholars have sought to develop overarching conceptualisations of the migration process, focusing on concepts such as ‘aspiration, desire and drivers’ of migration (Carling & Collins, 2018), the division of migration decisions into a ‘two-step process’ (Aslany et al., 2021; de Haas, 2021) or specific instances in which understanding of interactions between migration drivers might aid analysis of migration under particular circumstances (Black et al., 2011). Yet however much such contributions seek to challenge binary thinking and focus on the interaction of different factors influencing migration, the tendency remains to conceptualise migration as an event or ‘decision’, in which there may be a greater or lesser degree of agency or ‘choice’, and a greater or lesser influence of one of a range of contextual factors or ‘drivers’ (Ruedin, 2021).

The notion of ‘decision-making’ or ‘choice’ around migration is central to policy conceptualisations of migration as well. Indeed, there is now a long track record of policy interventions which seek to influence migration choice. These include classic information campaigns that work on the problematic premise that would-be migrants will make better choices—often implicitly not to migrate—if they have more or better information about destinations (Pécoud, 2010); through to more recent interventions that seek to widen choices locally or nationally, for example involving training or job creation for would-be migrants. Such interventions often, but not always, rest on a notion of migrants as *homo economicus*—although as we show below, governments and international agencies have become increasingly adept at appealing also to aspiration, culture and emotion. But what these interventions usually have in common is a notion of a ‘correct’, or at least better-informed choice that can result when public policy either influences or provides incentives, or encourages and supports certain actions.

The purpose of this paper is to interrogate this shared understanding of academic literature in migration studies that accepts the logics of migration policy as something that involves a decision or choice. Our approach is not to deny the significance of human agency in migration, or to say that migration does not involve a series of choices made by individuals and institutions. On the contrary, numerous studies point to the active engagement of migrants, their families and wider networks in the migration process (Massey, 2020). Rather, our aim is to propose an alternative understanding of what choice means in the context of contemporary migration in the region. We argue that choice is a more complex concept in practice than is often represented in ‘migration theory’, and that we need a more diverse, dynamic and future-orientated notion of choice as a lens for understanding when, why and how people migrate. Taking such a future-orientation seriously has profound implications however: as it points to uncertainty as the norm.

The analysis in this paper draws on research carried out within the context of the ‘Safety, Support and Solutions: Phase 2’ (SSSII) programme funded by DFID (now FCDO) and implemented by IOM and other partners, which ran from 2017 to 21.^1^ The project that enabled our data collection was aimed not at evaluating SSSII but at building from it to try to understand mobility decisions in a context in which much of West Africa experiences interventions aimed at development broadly understood.^2^ An interdisciplinary team comprising geographers, anthropologists and political scientists worked alongside the broader SSSII programme guided by an ethnographic research design. Fieldwork was conducted by the authors and a small group of research assistants, between July 2019 and March 2021. The sites selected ranged from the peripheries of the three capital cities of Dakar, Banjul and Conakry to larger towns such as Louga (Senegal), Kerewan (The Gambia), Boké and Kankan (Guinea), as well as rural sites dominated by agriculture and, in the case of Guinea, artisanal mining around the town of Siguiri. Selection of a total of 15 sites was guided by the careful avoidance of places where our funders would have active interventions in progress as it was imperative that the research was not associated with such interventions. For our ethnography, we concentrated on interventions in the area of livelihood and skills building for employability. We set out to conduct interviews with young people, community leaders, NGO and government officials across these 15 sites in Senegal, Guinea and The Gambia, as well as interviews with international organisations and national aid agencies. Across most of these sites, work drew on previous long-term ethnographic engagement of each field team which had explored related issues over the past two or more decades. We were thus able to draw on established contacts and friendships. Although disrupted by the 2020–21 Covid-19 pandemic, a field presence was retained in Guinea and Senegal throughout, and face-to-face interviews were supplemented with interviews via WhatsApp. Overall, we talked to 523 individuals. Designation of characteristics in the context of local villages and communities are blurred. For example, an interviewee might have been a young person, under-employed and a community leader. For this reason we have avoided more fine grained characterisations and instead focus on the ‘atmosphere’ or ‘discursive truths’ that are real in our communities. Specifically, interviews focused on people’s livelihoods and aspirations, as well as their experience of development interventions and migration within and beyond the region. The interviews ranged in approach from more formal direct questions to thematic conversations. We also undertook archival and documentary data collection, mainly from governmental and international organization sources focussed on migration, youth and labour policy areas. For this article we engaged in an iterative qualitative reading of our data to understand the perspectives of those we engaged with; however, without making claims of inductive generalization.

What transpired has informed the roadmap for our discussion below, where—in a next step—we engage with the literature to discuss relevant concepts and situate ourselves and read with and against what we have learned. Towards the latter half of this section we begin to bring in some of our data drawing on documentary and archival research, as well as interviews with international actors. Section three offers the ethnographic depth to problematise choice and turn our perspective to more productive engagement with how people live the present day and imagine their future. The section is organised by drawing out specificity from the different field sites in our three countries. Finally, we then turn to offering an interpretation and critical reading of what we have learned. We explore a futures lens, highlighting the creative projects of becoming with which people are engaged, despite often challenging conditions. This is important not only because policy-practice is so often ill-conceived and problematically practiced; but also in making a wider contribution to how we think and discuss movement across time, space and changing life projects.

## Migration drivers and migration choices

### Conceptualising the ‘migration decision’

Here is not the moment to delve into a detailed history of the development of ‘theories of migration’—this has been attempted numerous times before (Castles, 2010; Massey & Zenteno, 1999). In so far as a consensus has emerged, it has been around acceptance of the complexity of migration. Whether people move or not, they are influenced in their day-to-day decisions and actions, as well as in their longer-term approaches to life, relationships and society, by a complex set of economic, social, cultural, political and environmental factors. In particular, the reductive notion of *homo economicus* was perhaps always an ideal type—a device used by economists to strip away non-economic factors and to enable economic techniques to be brought to bear on understanding economic decisions and actions. Few would deny that such economic factors are real; equally, few would assert that they ever operate in isolation, and increasingly attention has been paid to the interplay between different factors (Haug, 2008).

What is common, however, to many theories of migration is the notion that migration itself involves a decision to migrate. Classically, that decision might be considered as a fundamentally voluntary one, which is amenable to analysis at either the micro or macro-level in terms of the motivations, values and expectations of individuals or social groups (De Jong & Gardner, 1981). By contrast, there are clearly some situations in which mobility is largely or wholly ‘forced’ by circumstances that compel an individual to leave. Such a distinction is an important one at the heart of public policy debates about ‘refugees’, typically from political persecution, but in recent decades also including those ‘forced’ to move by social, economic or environmental pressures, who are seen typically as having the ‘decision’ taken out of their hands. Yet even in the most ‘forced’ of circumstances, some may still ‘choose’ not to move, preferring escape to some local hiding place to potentially permanent displacement (Lubkemann, 2008). The possibility of choice remains.

On the other hand, simply deciding to move does not mean that an individual can necessarily do so even where there is a much greater degree of voluntariness. This observation underpinned the focus of Carling (2002) on distinguishing ‘aspiration’ and ‘ability’ to migrate, and is reiterated in the focus of Aslany et al. (2021) on a range of specific factors that appear from a systematic review of the literature to influence aspiration to migrate, including youth, ties to current or former migrants, dissatisfaction with public services and exposure to violence and insecurity. However, such a two-step approach still involves an implicit or explicit expectation that there is some choice, and a decision-making process of some kind (Carling & Schewel, 2018).

As part of a useful effort by Carling and Collins (2018) to bring theoretical rigour to the field, Van Hear et al. focus on ‘migration drivers’ in which people ‘choose to move … or have a decision thrust upon them’ (Hear et al., 2018: 927). Whilst the focus is on ‘drivers’ that limit or shape choice, the assumption again still remains that there is a decision—one made in the context of such drivers, and which might even influence them, but a decision nonetheless. Castles (2000) focuses attention on the ‘causes’ of migration which he locates more in globalisation, and global disparities of income, employment, social well-being or fertility. But again, he still refers straightforwardly to migration ‘decisions’ that ‘may be made not just by individuals’ but also as part of ‘family strategies’ (Castles, 2000: 270).

Erdal and Oeppen (2018: 985) thoughtfully take the discussion on choice further, noting the connotation of voluntariness in the context of options and alternatives. Whatever a decision is, it is only meaningful when the ‘… starting point for understanding volition in migration is the range and quality of alternatives available …’. In turn, one of the implications of thinking about migration as a process is to highlight the point that migration choices are unlikely to simply happen at one point in time. In the special issue on migration theories referred to above, Carling and Collins (2018) note how a range of authors seek to present migration in a way that is more ‘temporally distributed’, involving orientations to the future, and migration processes that are on-going through the life course. Thus Efionayi and Piguet (2014) describe how amongst prospective student migrants from Senegal, Côte d’Ivoire and Niger, many have proactive and well-researched strategies that are orientated to future careers.

### International interventions to influence migration choice

The relationship between migration decisions or drivers, and public policy, has also been a consistent theme in academic debate. In the intra-European context, De Jong and De Valk (2020) note the importance of welfare systems in influencing migration choices, but also note that these can often be conceived in quite ‘static’ terms. They call for attention to be paid to the relationship between migration decisions and the life course. There is also a rich vein of writing on the relationship between ‘voluntary’ return schemes for refugees and other migrants and both the decision-making process, and ultimate outcomes for returnees (Fennig, 2018; Webber, 2011). Yet such attention has been much less apparent in relation to public policy interventions designed to influence—normally to deter—migration between and from countries of the Global South. One such intervention, albeit not framed around deterrence, is the SSSII. This was framed as a ‘humanitarian’ intervention to provide support to migrants who became vulnerable once they had started migration across the Sahara to Europe, and also offered ‘sustainable reintegration support to help returnees rebuild their lives and deter risky remigration’.^3^

In practice, public policy interventions in this broad field are numerous, notably in connection with European Union policy (though not exclusively so), where the default assumption is that by promoting ‘development’, migration—to Europe at least—can somehow be averted or deterred. In the words of one of our interviewees at the regional level within an office in Abuja, EU and donor government funded activities are to ‘reverse migration’ by funding development rather than resorting to deportations. Building on the first Africa-Europe summit in 2000 in which migration was linked with development in Africa and freedom of movement called for within the African continent,^4^ the debate at the Seville Summit of the EU in 2002 was a landmark example in which migration from Africa to Europe, and Africa’s development were linked programmatically in terms of funding and technocratically in terms of capacity building. In this case, an initial proposal from UK Prime Minister Tony Blair and Spanish PM José María Aznar to make EU development aid conditional on strict controls on exit by African states was ultimately adjusted to focus instead on development aid designed to ‘incentivise’ lower migration, consistent with longstanding French approaches to ‘*co-développement*’.

Examples of such policies abound in West Africa, many of which have objectives expressed as offering economic alternatives to migration, with job creation, vocational training and support to entrepreneurs high on the list of interventions seen as offering a choice to young people not to migrate. This is particularly clear in relation to the operation of the European Union’s ‘Emergency Trust Fund for Africa’ (EUTF). The EUTF website talks of its “aim to establish inclusive economic development programmes addressing youths and vulnerable groups' employability through increasing their social and professional skills as well as enhancing effective job creation”, going on to say.

“Our main assumption is that short and long-term grievances arising from economic and social exclusion, marginalisation and inequality are amongst the most significant drivers of violence, forced displacement and illegal migration. Through the EUTF for Africa, we therefore aim to provide alternative opportunities for communities to foster growth and development in the long term”.^5^

In official communications, the EU and partners, such as the International Organization for Migration (IOM) highlight the ‘success stories’ of young entrepreneurs, often actually returnees rather than ‘potential’ migrants. Press releases heavily rely on eulogistic portraits of ‘successful’ beneficiaries, usually only one, sometimes two, with exemplary trajectories. In these portraits, the entrepreneurs are presented as brave characters who faced up to adversity to start their own business, with good prospects of a flourishing business,^6^ and who have ‘messages’ to deliver to young people like them: that self-employment is great,^7^ their country/region is great^8^ and the youth should not leave for Europe but should ‘return to the soil’. Female entrepreneurs are given special attention in these communications, although they are far from representing the bulk of beneficiaries. This is noteworthy as one ECOWAS official recounted how gender experts have not been engaged into the collaborative programming by UN agencies on migration, free movement and questions of labour market programming for young people until recently.^9^ Now women and their stories are pushed, neglecting that gender is not easily reducible to women alone.

Another example of such a programme is called ‘*Tekki Fii’*, which translates as ‘(you can) make it here’ in Wolof. This programme seeks to develop employment in Senegal by strengthening enterprise competitiveness and employability in ‘areas of departure’, specifically targeting ‘irregular migration’.^10^ The equivalent programme of the same name in Gambia offers grants, for example of 50,000 Gambian Dalasi (just under USD1000) for business to acquire equipment, licences and collateral. Again, the explicit framing is around the negative effects of irregular migration to Europe through the ‘backway’, and the need for economic alternatives. Interestingly, backway migration is also framed as ‘harming the country’s social cohesion and economy’,^11^ and the accompanying advertising campaign reflects an effort to appeal to culture and emotion, as much as economics. Thus as academic research debates choice vs. capability to migration, public policy narratives work instead on the assumption that ‘development’ interventions will allow the ‘choice’ not to migrate, even as the existence of this choice needs to be backed up with appeals to do the ‘right thing’ for the country.

A similar programme within the context of the SSSII was designed by a consultant with experience in the aid industry as well as in business. She recounted to us that she had originally approached programming with much the same assumptions: that young people *should* participate in entrepreneurship training; and if that training is well designed and implemented they will establish their own sustainable businesses and thus won’t want to migrate any more. Yet when the programme was piloted, many of those attending were absolutely clear that engaging with the training and even establishing a successful business would have little impact on their decision to migrate – should a situation arise where mobility was a necessity or even just a possibility, then mobility would be chosen.^12^ The implied conclusion that offering people the chance to reflect on business ideas and related skills is still a good idea, but it certainly is not a mechanism to fix people in place—at least not in the medium and long-term.

#### Future thinking, whole life approaches—the contribution of anthropology and African studies

As noted above, there has been some discussion already within the migration studies literature of the notion that migration decisions might be ‘temporally distributed’—in other words that decisions are not made at a single point in time, but that there is some level of future orientation, whilst decisions may be regularly returned to and adjusted through the life course. This reflects a broader movement within academic disciplines such as anthropology and geography to engage more substantively with thinking about the future—in ways that may be helpful as we seek to understand migration choices.

Indeed, the future has emerged as a substantial new field of study in the social sciences since 2000. Wars on terrorism and financial crises have produced a growing global atmosphere of uncertainty about the future; the Covid-19 pandemic crisis further accelerated this trend. The European governance crisis over migration from 2014 is sometimes fitted into such narratives about ‘crisis’. Social scientists, and anthropologists in particular, have documented and analyzed the impact of globalization on local communities and their sense of future and of time (Appadurai, 2013; Bear, 2014; Nielsen, 2014; Pandian, 2012; Piot, 2010). These studies often frame the future as an imagined and socially constructed horizon in relation to the inequalities of the present and the past. Meanwhile some also warn of potential future exploitation and domination: from macroeconomics to finance (Guyer, 2007; Ourossoff, 2010); from globalization processes to the question of aspirations (Appadurai, 2013; Carling, 2002; Collins, 2008); from urban to state planning (Abram, 2014); and towards the relationship between time and duration (Crapanzano, 2003; Hodges, 2008; Knight & Stewart, 2016; Morosanu & Ringel, 2016).

One area of the future in which anthropology has always shown interest, but which has also acquired new importance more recently are divinatory practices and prediction of the future (Malaby, 2003; Puri, 2015). Meanwhile in geography, concern with the future has a similar pedigree, from interest in anticipatory action (Anderson, 2010) to algorithms (Amoore, 2011) education (Kraftl, 2013) and anticipatory politics (Jeffrey & Dyson, 2020). And not only anthropology and geography have developed interest in the Anthropocene (Castree, 2014; Kirksey, 2018; Zee, 2017). The future is conceived as an object of aspirations, anticipations, speculations, projects and practices in a present that, in the end, largely reproduces itself and the past (Emirbayer & Mische, 1998). Conceiving of the future simply as the realisation of aspiration—as perhaps characterises existing literature on migration too—is not enough.

By contrast, the future as a transformative dimension is instead deeply intertwined with the notion of uncertainty, highlighted in African studies for example by a collection brought together by Cooper and Pratten (2015). Uncertainty is defined from this perspective as “the unpredictability and indeterminacy of what is going to be next, both immediately and in the long term. Living through uncertainty thus implies living in a state of contingency, that is, being dependent on courses of actions and events we cannot predict or know” (Di Nunzio, 2015: 152–53). Planning and *making* the future remains an open-ended effort (Di Nunzio, 2019), which keeps in circulation the possibility of redesigning or giving up one’s aspirations.

At the heart of the knot between future and uncertainty lies an experience of time understood as duration or social becoming. In this knot the threads of secular and religious, macro-economic, social-relational and ethical temporalities intertwine and contrast with each other. The practices and imaginaries of communities, and especially young people, then become arenas in which to study these repertories of the future, of action and responsibility, of dependence and freedom. As Bryant and Knight put it in a recent contribution:

“the future awakens the present through a plethora of orientations with indeterminate teleologies. We are constantly anticipating, expecting, hoping for, and speculating about—and thus living—the future in everyday life” (Bryant & Knight, 2019: 192)

The orientation towards the future represented in this approach reflects the different ways in which people prepare the ground for the future in the present (see also Engeler & Steuer, 2017). In this sense “the future is encountered in novelty and repetition, but also in aspiration and inertia. […] At the moment of (non-) decision and (in-)action, a projection or imagining of the future bubbles into the present and influences what we do.” (*ibid*: 198) In other words, as with other interventions throughout the decades, assumptions of linearity from cause to effect and the arrogance of assuming control—even if expressed via ‘incentives’—fall apart at the point when they hit the reality of the capacity of those intervened upon to imagine their own futures. Indeed, this capacity exists, in big and small ways and enacted by docility and inaction as much as by instant or deferred action, as potential.

## Migration and choice in West Africa

Some perspectives on West African migration have already opened up to the idea that the ‘cultures of migration’ and new mobility regimes have transformed and subverted social configurations and hierarchies, structures of kinship and affective ties, experiences of mobility and social redemption, repertories of the future and of hope (Kleist & Thorsen, 2017; Klute & Hahn, 2007). In an inversion of Carling’s notion of ‘involuntary immobility’, some work in the region has also focused on the notion of ‘existential mobility’—the sense of being socially on the move or going somewhere in life (Hage, 2009; Hage & Papadopoulos, 2004). Hage distinguishes between physical and existential mobility and describes the latter as ‘an intrinsic feature of how we experience our humanity’ (Hage & Papadopoulos, 2004, 112). According to Hage, one of the motivations for migration is a sense of existential stuckedness or of moving too slowly. Migration is a ‘powerful trope for imagining oneself as being socially on the move […] and a way of imagining a possible escape from a frozen future’ as Gaibazzi has noted in relation to young Soninke men in The Gambia (2014, 173). In other words, the urge to go somewhere in life (existential mobility) may be dealt with through physical mobility. Conversely, existential immobility or ‘stuckedness’ refers to the feeling of not going anywhere in life, of being stuck” (Kleist & Thorsen, 2017, 13–14). In an alternative formulation, Engeler (2019) talks of ‘meandering lives’ of young people in Guinea as they explore paths to adulthood that are both temporal and spatial.

Language matters when people try to express ideas and hopes. At one level, the idea of choice is routinely contrasted with that of ‘fate’—a concept that in predominantly Muslim societies across all three countries has wide currency. An alternative notion was that choice was described as ‘sweet’, and the absence of choice ‘bitter’. With the successive effects of structural adjustment, economic marginalisation, the Ebola crisis, and at the time of fieldwork, the severe economic effects of the Covid-19 pandemic, for many sweetness was rare, whilst bitterness was everywhere. We explore these themes, and the associated uncertainty and open-endedness of life prospects respectively in each of our case study countries below.

### “Put into a corner” on Gambia’s North Bank

International migration has a long history on the North Bank Division of The Gambia, dating at least to the colonial period when there was seasonal migration to the peanut growing areas of Senegal, and then migration to the diamond-fields of Sierra Leone in the 1950s. Today, migration shapes the panorama of rural villages of the region as much as representations of individual and family wellbeing. Local interlocutors, when asked about the diaspora, point to new construction under way inside family fences, and remark that money came from migrants abroad. According to a local young man from the village of Kerewan, the extended family is often a site in which the social pressure to migrate reaches its peak. Polygamous families are numerous in Kerewan and the children of junior wives feel that they are “*put into a corner*”—where they would do anything to equate or surpass the material contribution to the family of their elder siblings.^13^ Young men running businesses in the village of Kerewan reported that migration is lived, by the youth, as a prescription. Before opening a shop, one of them undertook the backway migration and described his trajectory in this way:

There were elders insulting us: ‘You are nothing! Look at your colleagues who leave to Europe. You are here sitting doing nothing’. So, I went through a period when I was frustrated. I said to myself: ‘What am I doing here in the country? My father is not alive, my mother is not alive. I am not married. I will also go’.^14^

At the same time, the local economy, based on agriculture, *needs* migration—whether in the form of international migration, or settlement in the urban and peri-urban coastal areas of The Gambia. For example, three businessmen hailing from Kerewan, who we met in Serrekunda, stressed the burden of providing the bulk of material contributions to their original households in the village. One of them expressed his duty to support his mother, stepmothers and younger brothers still in Kerewan:

That is a compulsory obligation. I help in fencing, digging of wells or pay people to do it. I also assist in buying seedlings and fertilizers. My people still do farming but my brothers do it and go to school while I focus on the business. Our father is now late.^15^

The interviewee was referring to horticultural activities of the household conducted by senior women, his mother and the co-wives. The women manage autonomously the income generated from horticulture to cover a part of the daily food expenses, buy clothes and organize family ceremonies such as marriages, naming ceremonies etc. Evidence from other parts of the country shows that, faced with lack of government subsides; farmers need either the diaspora or the family members with a regular income to finance the beginning of a new season. The debt-ridden situation of Gambian agriculture is an age-old problem that results from economic and political strategies put in place, since the 1850s, to turn the country and the region into a peanut-export zone (Brooks, 1975).

The North Bank, due to its historical connections with Senegal, is an interesting site also to consider the real and imagined barriers and potentials of commercialization and cross-border trading in the context of not-quite functioning ECOWAS freedom of movement policies. Between the late 1990s and the 2010s, the Jammeh government intervened to support strategic transport infrastructure in the country and this directly affected the mobility of the citizens in the country and within the broader region. Our interviewees in the provinces, the hinterland regions of The Gambia, highlighted the deep impact brought about by road refurbishment. The chairman of the Village Development Committee of Brikama Ba, Central River Region, explained that during the past two decades, connections with the coastal urban areas have drastically improved in terms of duration of the trip and availability of vehicles.^16^ This allows many more young people from the village to go to work to the coast. The young men we interviewed in the village of Mansajang, Upper River Region, have spent part of their lives on the coast where they undertook apprenticeship pathways; many of them moving back and forward, engaging in daily labour on the coast during periods of agricultural inactivity in his home village.^17^

There is not one single person who is not imagining to go ‘wander’ in the future; and when this imagining is combined with social pressure and with learning about being mobile over shorter distances and periods of time, dreams about what may be possible are tested and teased. Testing and teasing might lead to information gathering about how to move further afield, including how to go about trans-national and trans-continental mobility. This is how a journey starts: there is no point of decision; rather there are a myriad of ways to go from dreaming and imagining—living life in the future, rather than a concrete aspiration or plan—to enacting in the present whenever that becomes possible.

Throughout the past 20 years, what has been maintained is a public rhetoric of ‘making it in The Gambia’, in contrast to the ‘lure’ of migration. Indeed, both during the Jammeh period and after regime transition, the country’s national elites have openly criticised young peoples’ trans-continental mobility to Europe, while, at the same time, engaging actively in such mobility themselves, along with their close relatives. For example, official government missions to Europe and North America provided a path of capital accumulation for high-ranking civil servants and politicians, who—as a reaction to Jammeh’s volatility—drew down state resources as if there was no tomorrow.

Since 2017, international donors (especially the EU) have stepped in by funding projects deliberately targeting people who are defined on opaque criteria as ‘potential migrants’—a category that can be read as meaning all young people. Yet the ‘*Tekki Fii*’ project mentioned above drew widespread scepticism from our respondents—both from potential beneficiaries and from activists involved in the implementation. We saw a general disillusionment towards the role of both national and local elites in supporting the social and economic maturation of young individuals. A young businessman based in Kerewan commented:

[The youth] don’t believe they will make it in their country anymore. […] We don’t believe in our leaders, our government sector, anymore. You cannot go in a ghetto in these days without finding skilful youth… carpenters, painters, mechanics… but the reality is that they don’t believe in what they are doing… the society, in fact, is not believing in what you are doing, is not supporting what you are doing. You become foolish to yourself! You know… I have this small shop… the society I live in do not normally come and buy materials from me, they prefer to go to a foreigner. So if in two or three months I get discouraged, what am I going to do? Close the shop and go.^18^

This statement certainly reflects resentment, and often this is all it is—an expression of unhappiness, the indication that there is a plan B—quite possibly a diffuse plan B lacking any concreteness but a future-oriented imagining nonetheless. Sometimes, however, it might be acted upon. Doing so within the ECOWAS region is, at least in principle, governed by provisions of freedom of movement, and in practice, four fifths of all mobility is such regional mobility. Nonetheless there is a widespread discourse in the country that migration equates with going to Europe or America, one that was regularly referred to in interviews, and that is reinforced by development interventions such as *Tekki Fii*.

### “Stay and develop your land” in rural Senegal

As in The Gambia, agriculture was and remains the mainstay of the Senegalese rural economy, and there is a similar relationship between agriculture and migration. Here too, the importance given to farming has prompted interventions since before and after independence: for example, after independence, public authorities set up development and operating companies such as the *Société de Développement Agricole et Industriel du Sénégal* (SODAGRI) to develop the valleys for rice-growing purposes and a financing and technical support company—*Société de Développement et des Fibres Textiles du Sénégal* (SODEFITEX)—for cotton production, whilst more recently there are interventions aimed at promoting young people’s ‘return to the land’ such as Anida’s TV reality show ‘Ferme factory’, which is co-funded by the EU.^19^ Initially, such interventions were welcomed in rural areas and held out much hope. But the euphoria did not last long, as they did not produce the hoped-for results for local people. Indeed, in the Amnabé basin, government action through hydro-agricultural developments is seen as the catalyst for food insecurity, as one of our respondents reported:

"The developed areas have resulted in monoculture. Before, people used to grow corn, groundnuts, rice, beans, etc.... All the land that used to be used for crop diversification is now allocated to rice production with the hydro-agricultural developments. It is no longer possible to grow several crops [...]. Rice cultivation does not meet the food needs of many families. You find many families who don't have enough to eat because rice production is not working" (GS, Diaobé, May 2020).

Food insecurity and disillusionment around agricultural development interventions, family pressures and the lack of viable alternatives at the local level mean that young people feel they have no other option but to migrate in search of well-being:

"I, who had invested 200,000 FCFA [in agriculture], I harvested nothing but a small bag of rice. It's a wasted effort, since then I haven't been farming. That's why we young people don't see each other anymore in agricultural activities and we try to go elsewhere" (AK, Diaobé, March 2020).

The commune of Diaobé-Kabendou was one of 23 across Senegal to benefit in 2010 from the creation of the “Young People on Farms” project, which provided training to 15 young people to farm an area of around 5 ha. Supervised by the Ministry of Youth, the project formed part of the *Grande Offensive Agricole pour la Nourriture et l’Abondance* (GOANA) launched by President Wade in his second term in 2008, and benefitted from equipment such as seed drills, soil disinfection and fertilisation equipment, carts and other agricultural machinery. Yet due to a lack of follow-up and sufficient means, the project was unable to retain the young people involved.

"The young people with whom we share the project went to Europe [...]. They are at least five who have left for Europe. Because they did not see what they expected from the project" (AS, Diaobé, April 2020).

Another project was run by the Italian NGO *Fondazione ACRA*, the University of Milano-Bicocca and the *Association Guné de Kolda*, supported by the Italian Agency for Development Cooperation in 2017. The association "Guné" and its partners chose as their slogan: "Stay and develop your land" or in Pulaar "*Heddaade e Bamtaade do Mun*". Awareness raising and the creation of employment opportunities at the local level were seen as the levers for action. Awareness-raising was centred around the market and certain villages. Music concerts by nationally and internationally renowned artists, home visits, focus groups, film screenings with debates, iterative animation sessions with the public, sketches etc. were carried out to raise awareness about the dangers of illegal emigration. The project leaders financed the development of market gardening areas, chicken coops and the processing of local products for the benefit of youth and women's associations. Yet respondents reported that the chicken coops and mills financed by the project quickly went bankrupt when the project ended. One former project manager described the region as a ‘graveyard of projects’, where young people are interested to engage in ‘projects’ as an opportunity, but not in the activity they are in. Meanwhile returnees who had joined the project had moved again, including back to Europe, to secure livelihoods. Here is an example of how imagining the future is a way to make life happen: those who had ‘benefitted’ from interventions to help them stay locally had never stopped imagining a possible life far away. But as migration is framed as a bad thing, we are blinded to what it can offer: learning and experience is both moving through time and through space, food in the belly and hope is also certainly both temporal and spatial. How would it be if people could imagine their future as a migrant with joy and pride, rather than tinted by resentment of all sorts?

### “Cultivate and marry” in rural and urban Guinea

Of course not all development projects fail, nor is agricultural success always out of reach. In our field sites in the Kankan region of Guinea, agricultural cooperatives sometimes provide more secure routes to a stable income, and the notion that “the land keeps”—farming land will allow people to stay in their local areas—is more persuasive. One interviewee had helped organise and fund a friend’s migration project to Europe, with the idea that the friend would send money to help him expand his market gardening activity and improve productivity. In this case, although the friend did not keep his promise, he had been able to complete the building of the house that his father had started, and provide for a large family. Indeed, this individual was well-known, and cited by local peasant associations as an example of how through market gardening, it is possible to continue to live in Guinea without being forced to go abroad. Nonetheless, in due course, some of his own children had migrated—in this case to Conakry or to other African countries.

Whilst examples exist in Guinea too of interventions that do not last beyond the period of project funding, a counter example funded by IFAD is of interest. In this case, literacy training provided by a cooperative used N'ko, a local alphabet created to be able to write all the sounds of the Malinké language. Under a small shed, women gathered in front of a blackboard where the trainers teach them how to write in N'ko. "This helps us a lot," the president of the KOMISAKA group, a woman over 60 years old, told us:

"Thanks to Nko, the women can write the agricultural calendar and plan their activities. We also learn how to calculate, prepare our budget, forecast expenses and yields. They see literacy in N'ko as an alternative to French, the official language of Guinea, which is much more distant from their daily experiences. It saves time, as it is easier for women used to speaking Malinké to understand the notions of time and measurement if it is explained in N'ko.”

Nonetheless, the notion of ‘choice’ remained rare amongst interviewees. Training, for example, was rarely a ‘choice’ for young respondents, but a consequence of the closing off of other routes, notably educational success, and a way to open up other possible futures. And whilst migration was sometimes framed by those in agricultural cooperatives as an individual choice, compared to ‘development’ as a collective endeavour, this was not always how it was seen by individuals themselves.

For example, interviewees in the capital Conakry often reacted with surprise when asked about migration choices. Most reported being mobile in their past life and expected to be mobile again in the future, whether to go to school or college in another setting, to spend a couple of years at the home of a relative, or because of (potential) work opportunities elsewhere. Geographical mobility was seen very much as related to social mobility (Botta & Somparé, 2018) and this geographical mobility included moving between rural and urban settings but also going beyond national borders, including to Europe. Indeed, migration is not perceived as something special or extraordinary. In particular during childhood, during the often prolonged youth, but also during adult life, Guineans do move. And contrary to the depiction within cooperatives noted above, the migration often depends not on the individual but on the broader family, following pathways paved by relatives or as a reaction to family affairs.

An example is provided by a young woman interviewed in Ratoma, Conakry, who a couple of years previously had lost her father, could not continue with school and was facing forced marriage. How could she support her now widowed mother and her younger siblings? Was becoming the second wife of a much older man living upcountry the only option? Or was it time to go with one of her female friends who offered to accompany her, and even support her financially, if she were to leave Conakry with her to look for greener pastures elsewhere? In fact, she did leave Conakry to try her luck beyond national borders; but while dreaming to reach Europe she experienced only demanding and dangerous months on the road to finally get stuck in Algiers. After months of precarity and casual work she was robbed and decided to go home—with the assistance of a repatriation programme—where she received further assistance and started a training program at a tailoring workshop.

At the time of research, this respondent lived with her mother again and earned enough money to from time to time support her siblings. She dreamt of establishing her own atelier and marrying soon—she had two potential candidates in mind already. Thus, she had managed to create alternative futures or perspectives while re-establishing herself and for now remaining in her home town. Yet she also described life as very uncertain and knew that at another point in her life, migration could again be an option. In her case, her future life trajectory strongly depends on her economic independence, her marital status and on socio-political circumstances shaping Guinea society more generally. As Johnson-Hanks wrote about young Cameroonian women; “life is so uncertain that plans are always tenuous, partial, more hope than conviction” (Johnson-Hanks 2016: 12). Thus at vital conjunctures in life, “multiple potential futures are in play” (Johnson-Hanks 2016: 7)—for instance becoming the second wife of an unknown vs. becoming mobile. In this context, migration is neither the norm, nor an exception, but one of a set of alternative imaginations of the future.

## Discussion and conclusion

This paper has set out to explore some of the factors surrounding mobility in West Africa by scrutinizing ‘choice’, when individuals are faced with development interventions designed to influence that choice. The region is an interesting place to do this, given the significance of mobility for people imagining their futures—both within the ECOWAS region, but also in Europe. The extent and diversity of interventions that have sought to engage with migration over the past decade have also markedly increased in the region, but mirror those elsewhere. West Africa is perhaps unusual in the extent to which an emic culture of migration exists in the region, based on a long history of mobility, and the wide variety of national and regional destinations, and this places some limits on the scope for generalisation. That said, it is worth noting that worldwide, migration within countries and across borders to neighbouring countries numerically far outweighs longer-distance migration, and yet still is often ignored in public and policy discourses.

What we have seen is that in Guinea, Senegal and The Gambia at least, whether people migrate or not, or indeed where they migrate, is often not exactly a choice—but equally not a lack of human agency, or options. In this context, public policy framed to try to widen choice—to provide alternatives to migration—often falls flat, because it has misunderstood how migration and mobility intertwine with life trajectories that are frequently uncertain and unpredictable. An obvious and key element of development interventions based on ‘expanding choice’ is the assumption that choices exist, whilst at the same time limiting it to the binary of ‘to migrate or not to migrate’, for example, with this binary implicitly eliding ‘migration’ with ‘migration to Europe’. Yet when we cannot have any certainty about the future, what does it mean to make life choices, whether these are economic, cultural or emotional?

Of course there is a more practical element to the cases revealed at our different field sites. Some interventions conceptualised to increase choice and so reduce migration fail simply because projects are poorly conceptualised, or are dependent on external resources for impact, and so cannot outlast the period of project intervention. Development interventions also—like all interventions—have unintended and complex consequences. Although rarely framed as an intervention to address migration, the current emphasis in West Africa on road building as part of many development interventions can be conceptualised both as indirectly improving local development options, and so potentially reducing migration, but also directly increasing individuals’ opportunities to migrate, especially regionally, for example through opening up long-distance coach travel. Equally, involving the diaspora in job creation and training activities also ironically showcases the benefits of migration, in particular trans-continental migration, as one EU official commented during interview.^20^

In pointing attention to the need to understand West African migration through a ‘future-orientated’ lens, that emphasises a notion of ‘becoming’ rather than a moment (or stages) of choice, we are not asserting that migrant agency is absent, nor indeed that migration and development policy is irrelevant or impossible. But this approach does draw attention to the at best irrelevance, and at worst damaging nature of public policy that seeks to fix young people in place. More important, in an inversion of the common western notion of the future, for many of our respondents it is the present that is uncertain and full of risk, and the future that offers more certainty and hope. Migration choice is not a two-stage process, leading from aspiration to ability to migrate—the former certain but the latter uncertain; rather it is an ever-present imaginary of an implausible but also dazzling future that is waiting to emerge from the chaos of the present.

## Data Availability

The study is based on qualitative interview materials which were collected on the basis that they remain confidential. They are stored in anonymised format on a secure server at the university of the corresponding author.

## Footnotes

1 https://devtracker.fcdo.gov.uk/projects/GB-GOV-1-300461.

2 https://www.birmingham.ac.uk/schools/government/departments/international-development/research/projects/2019/migchoice/about.aspx.

3 https://africa-eu-partnership.org/sites/default/files/pres-00-901_en_0.pdf. Accessed 15 Feb 2021.

4 https://africa-eu-partnership.org/sites/default/files/pres-00-901_en_0.pdf. Accessed 15 Feb 2021.

5 https://ec.europa.eu/trustfundforafrica/thematic/greater-economic-and-employment-opportunities.

6 https://ec.europa.eu/trustfundforafrica/all-news-and-stories/absa-jallow-recounts-her-migrant-returnee-experiences-and-expresses-hope-better_en.

7 https://ec.europa.eu/trustfundforafrica/all-news-and-stories/le-message-dalimatou-ses-compatriotes-senegalais_en.

8 https://ec.europa.eu/trustfundforafrica/all-news-and-stories/cest-avec-son-hotel-en-casamance-que-pascal-ehemba-pu-realiser-son-reve_en.

9 Interview, Abuja ECOWAS headquarters, social affairs division, March 2020.

10 https://www.acted.org/fr/developper-lemploi-avec-tekki-fii/.

11 https://yep.gm/about.

12 Interview, IOM consultant on IOM outcome 4 youth project, February 2021.

13 Interview in Kerewan, February 2020.

14 Interview in Kerewan, March 2020.

15 Interview in Serrekunda, March 2020.

16 Interview in Brikima Ba, February 2020.

17 Interviews in Mansajang, February 2020.

18 Interview in Kerewan February 2020.

19 https://www.jeuneafrique.com/mag/899749/culture/ferme-factory-une-emission-de-tele-realite-senegalaise-pour-promouvoir-le-metier-dagriculteur/.

20 Interview at EU delegation, Dakar, September 2020.

## References

[CR1] Abram S (2014). The time it takes: Temporalities of planning. Journal of the Royal Anthropological Institute.

[CR2] Amoore L (2011). Data derivatives: On the emergence of a security risk calculus for our times. Theory, Culture and Society.

[CR3] Anderson B (2010). Preemption, precaution, preparedness: Anticipatory action and future geographies. Progress in Human Geography.

[CR4] Appadurai A (2013). The future as cultural fact.

[CR5] Aslany, M., Carling, J., Mjelva, M. B., & Sommerfelt, T. (2021). *Systematic review of determinants of migration aspirations.* QuantMig deliverable 2.2. Southampton: University of Southampton.

[CR6] Bear L (2014). Doubt, conflict, mediation: The anthropology of modern time. Journal of the Royal Anthropological Institute.

[CR7] Black R, Bennett SRG, Thomas SM, Beddington JR (2011). Migration as adaptatoin. Nature.

[CR8] Botta, E., & Somparé, A. W. (2018). Le nuove identità di Conakry. In E. Botta, & A. W. Somparé (Eds.), *L'Africa delle città/Urban Africa*. Torino: Academia Press.

[CR9] Brooks GE (1975). Peanuts and colonialism: Consequences of the commercialization of peanuts in West Africa, 1830–70. Journal of African History.

[CR10] Bryant R, Knight DM (2019). The anthropology of the future.

[CR11] Carling J (2002). Migration in the age of involuntary immobility: Theoretical reflections and cape Verdean experiences. Journal of Ethnic and Migration Studies.

[CR12] Carling J, Collins F (2018). Aspiration, desire and drivers of migration. Journal of Ethnic and Migration Studies.

[CR13] Carling J, Schewel K (2018). Revisiting aspiration and ability in international migration. Journal of Ethnic and Migration Studies.

[CR14] Castles S (2000). International migration at the beginning of the twenty-first century: Global trends and issues. International Social Science Journal.

[CR15] Castles S (2010). Understanding global migration: A social transformation perspective. Journal of Ethnic and Migration Studies.

[CR16] Castree N (2014). The anthropocene and geography I: The back story. Geography Compass.

[CR17] Collins J (2008). "But what if I should need to defecate in your neighbourhood, Madame?" Empire, redemption, and the "tradition of the oppressed" in a Brazilian world heritage site. Cultural Anthropology.

[CR18] Cooper E, Pratten D (2015). Ethnographies of uncertainty in Africa.

[CR19] Crapanzano V (2003). Imaginative horizons: An essay in literary-philosophical anthropology.

[CR20] de Haas H (2021). A theory of migration: the aspirations-capabilities framework. Comparative Migration Studies.

[CR21] De Jong GF, Gardner RW (1981). Migration decision making: Multidisciplinary approaches to microlevel studies in developed and developing countries.

[CR22] de Jong P, de Valk H (2020). Intra-European migration decisions and welfare systems: The missing life course link. Journal of Ethnic and Migration Studies.

[CR23] Di Nunzio, M. (2015). Embracing uncertainty: Young people on the move in Addis Ababa's Inner City. In E. Cooper, & D. Pratten (Eds.), *Ethnographies of uncertainty in Africa* (pp. 149–72). London: Palgrave Macmillan.

[CR24] Di Nunzio M (2019). Not my job? Architecture, responsibility and justice in a booming African metropolis. Anthropological Quarterly.

[CR25] Efionayi D, Piguet E (2014). Western African student migration: A response to the globalisation of knowledge. International Development Policy.

[CR26] Emirbayer M, Mische A (1998). What is agency?. American Journal of Sociology.

[CR27] Engeler, M, & Steuer, N. (2017). Elusive futures: An introduction. In N. Steuer, M. Engeler, & E. Macamo (Eds.), *Dealing with elusive futures: University graduates in urban Africa* (pp. 9–25). Bielefeld: Transcript Verlag.

[CR28] Engeler M (2019). Youth and the state in Guinea: Meandering lives.

[CR29] Erdal MB, Oeppen C (2018). Forced to leave? The discursive and analytical significance of describing migration as forced and voluntary. Journal of Ethnic and Migration Studies.

[CR30] Fennig M (2018). Beyond voluntary return: A critical ethnographic study of refugees who departed Israel 'voluntarily'. International Social Work.

[CR31] Gaibazzi P (2014). Visa problem: Certification, kinship, and the production of 'ineligibility' in the Gambia. Journal of the Royal Anthropological Institute.

[CR32] Guyer JI (2007). Prophecy and the near future: Thoughts on macroeconomic, evangelical and punctuated time. American Ethnologist.

[CR33] Hage, G. (2009). Waiting out the crisis: on stuckedness and governmentality. In G. Hage (Ed.), *Waiting* (pp. 97–106). Carlton: Melbourne University Publishing.

[CR34] Hage G, Papadopoulos D (2004). Ghassan Hage in conversation with *Dimitris papadopoulos*: Migration hope and the making of subjectivity in transnational capitalism. International Journal for Critical Psychology.

[CR35] Haug S (2008). Migration networks and migration decision-making. Journal of Ethnic and Migration Studies.

[CR36] Hear V, Nicholas OB, Long K (2018). Push-pull plus: Reconsidering the drivers of migration. Journal of Ethnic and Migration Studies.

[CR37] Hodges M (2008). Rethinking time's arrow: Bergson, Deleuze and the anthropology of time. Anthropological Theory.

[CR38] Jeffrey C, Dyson J (2020). Geographies of the future: Prefigurative politics. Progress in Human Geography.

[CR39] Kirksey E (2018). Queer love, gender bending bacteria, and life after the Anthropocene. Theory, Culture and Society.

[CR40] Kleist N, Thorsen D (2017). Hope and uncertainty in contemporary African migration.

[CR41] Klute, G., & Hahn, H. P. (2007). Cultures of migration: Introduction. In H. P. Hahn, & G. Klute (Eds.), *Cultures of migration: African perspectives* (pp. 9–30). Berlin: Lit Verlag.

[CR42] Knight DM, Stewart C (2016). Ethnographies of austerity: Temporality, crisis and affect in southern Europe. History and Anthropology.

[CR43] Kraftl P (2013). Geographies of alternative education: Diverse learning spaces for children and young people.

[CR44] Lubkemann SC (2008). Involuntary immobility: On a theoretical invisibility in forced migration studies. Journal of Refugee Studies.

[CR45] Malaby TM (2003). Gambling life: Dealing in contingency in a Greek City.

[CR46] Massey D (2020). Immigration policy mismatches and counterproductive outcomes: Unauthorized migration to the U.S. in two eras. Comparative Migration Studies.

[CR47] Massey DS, Zenteno RM (1999). The dynamics of mass migration. Proceedings of He National Academy of Sciences.

[CR48] Morosanu R, Ringel F (2016). Time-tricking: A general introduction. Cambridge Journal of Anthropology.

[CR49] Nielsen M (2014). The negativity of times: Collapsed futures in Maputo, Mozambique. Social Anthropology.

[CR50] Ourossoff A (2010). Wall street at war: The secret struggle for the global economy.

[CR51] Pandian A (2012). The time of anthropology: Notes from a field of contemporary experience. Cultural Anthropology.

[CR52] Pécoud, A. (2010). Informing migrants to manage migration? An analysis of IOM's information campaigns. In M. Geiger, & A. Pécoud (Eds.), *The politics of international migration management* (pp. 184–201). Basingstoke: Palgrave Macmillan.

[CR53] Piot C (2010). Nostalgia for the future: West Africa after the cold war.

[CR54] Puri SS (2015). Betting on performed futures: Predictive procedures at Delhi racecourse. Comparative Studies of South Asia, Africa and the Middle East.

[CR55] Ruedin D (2021). Decision-making under uncertainty. African migrants in the spotlight. Social Inclusion.

[CR56] Webber F (2011). How voluntary are voluntary returns?. Race and Class.

[CR57] Zee JC (2017). Holding patterns: Sand and political time at China's desert shores. Cultural Anthropology.

